# Study on the resistivity characteristics and mechanism of silt clay under different initial conditions

**DOI:** 10.1371/journal.pone.0319072

**Published:** 2025-04-02

**Authors:** Chundong Wang, Fuming Bao, Youqian Lu

**Affiliations:** 1 Powerchina Huadong Engineering Co., Ltd., Hangzhou, China; 2 Guangxi Transportation Science and Technology Group Co., Ltd., Nanning, China; 3 School of Civil Engineering, Beijing Jiaotong University, Beijing, China; Kielce University of Technology: Politechnika Swietokrzyska, POLAND

## Abstract

By taking silt clay as the research object, two-phase electrode resistivity tests under different water content and dry density conditions were carried out to clarify the resistivity variation law and influence mechanism of silt clay. The results show that the resistivity of the soil decreases sharply in the low moisture content section then tends to stabilize with the change of moisture content, and decreases continuously with the increase of dry density. There are three phases of a medium, namely soil, water and air, in unsaturated soil, so there are mainly three conductive paths: between soil particles, between pore fluids, and between soil-water coupling. Under different moisture content and dry density conditions, there are obvious differences in the effective contact area, and the types and numbers of conductive paths, which in turn affect the resistivity of the soil. The water status and pore structure of the silt clay samples were analyzed by hydrogen nuclear magnetic resonance (1H-NMR) results to clarify the conductive mechanism of unsaturated silt clay. Finally, a volumetric moisture content and resistivity model is established to unify the effects of moisture content and dry density on resistivity, providing a theoretical reference for the construction and operation safety of silt clay engineering.

## Introduction

With the rapid development of industrialization and urbanization in China, engineering construction is also constantly increasing, and more and more engineering construction will be aimed at areas with silt distribution. Silt is subject to seasonal climate change, which causes periodic dry-wet cycles and bulking and shrinkage deformation, resulting in different moisture content, density and structure of the soil [1].

Soil electrical conductivity is a critical indicator for assessing soil properties such as moisture content, salinity, strength characteristics, pore structure, and the concentrations of heavy metals or other chemical substances [2,3]. Moisture provides a medium for ion migration, while salinity increases the ion concentration in the solution, both of which affect soil electrical conductivity. Additionally, pore structure influences ion transport pathways, and heavy metals or other chemical substances alter the ionic composition of the soil solution, thereby impacting soil electrical conductivity. Soil electrical conductivity is highly sensitive to these soil parameters and offers advantages such as being non-destructive, rapid, and capable of continuous measurement. With increasing human activities and growing environmental concerns, this focus has become particularly significant in the context of environmental monitoring and pollution assessment. Understanding changes in soil electrical conductivity can aid in identifying pollution sources, guiding remediation efforts, and providing valuable insights for evaluating soil health and potential risks to ecosystems and human activities [2–4]. The measurement and estimation of soil electrical conductivity are crucial for engineering design and the evaluation of soil engineering properties. For instance, parameters such as soil strength characteristics, consolidation properties, moisture content, and solute transport can be determined through soil electrical conductivity. These parameters are essential for calculating the safety factor of slope stability, controlling geomembrane failures in landfills, ensuring the quality of compacted soil liners, assessing soil-bearing capacity, and evaluating engineering risks related to solute transport in groundwater [5,6]. Therefore, understanding the factors influencing soil electrical conductivity and its variation is of great significance for accurately evaluating soil electrical conductivity, predicting soil behavior, and assessing its environmental impacts [7,8].

The values of resistivity and electrical conductivity are inversely proportional and can directly reflect the electrical conductivity characteristics of soil, which is one of the most difficult parameters to estimate, because several factors such as mineral content, grain size distribution and shape, permeability coefficient, ambient temperature, moisture content and porosity have a strong influence on soil properties [9–11]. Currently, many researchers have investigated the influence of soil moisture content on soil resistivity and explored the functional relationship between the two [12–15]. Hassona et al. [16] found that there is a correlation between resistivity and moisture content, liquid and plastic limits, and clay content through physical-mechanical and electrical experiments. Resistivity can be used as a representative parameter for characterizing the engineering properties of soil. Siddiqui et al. [17] investigated the relationships between resistivity and various soil properties, including moisture content, strength parameters, effective size *d*_10_, and liquid and plastic limits. Their study revealed that different equations were derived for each soil type, with no universal equation capable of encompassing all experimental results.

Apart from the influence of the aforementioned soil properties, the state of pore water also has a significant impact on resistivity. Samoulian et al. [18] have shown that the resistivity of soil is mainly influenced by external environmental factors such as the temperature of the soil, the medium environment, and some important structural indicators of soil such as saturation, porosity, pore shape, and the resistivity of the solution inside. The electrical conductivity of pore water in soil largely determines the overall soil electrical conductivity, so the moisture content and saturation have a significant impact on the strength of soil electrical conductivity [3,19]. McCarter [20], Kalinski [21], and Abu Hassanein, et al. [22,23] conducted resistivity tests on remolded soils, analyzing the influence of state variables such as porosity, saturation, and moisture content on resistivity. They also investigated the correlation between resistivity and permeability. The results indicated a distinct relationship between resistivity and initial (compacted) saturation, independent of compaction effort. However, the relationship between resistivity and hydraulic conductivity was not unique, suggesting that resistivity testing is unlikely to replace hydraulic conductivity testing. McNeill [24] defined critical saturation as the minimum saturation at which the water film in the soil maintains continuous penetration. When the saturation of the soil is less than its critical saturation, the conductivity of the soil will suddenly decrease due to the obstruction of current propagation along the path of water in the soil.

Some scholars eliminated the influence of other influencing factors on resistivity through parametric analysis, normalization analysis and corresponding test measures, and obtained a direct correlation between resistivity and soil microstructure [25–28]. Fukue et al. [29] conducted laboratory resistivity tests to explore the relationship between soil conductivity and microstructural characteristics. The experimental results indicated that the relationship between soil conductivity and water content can be used to calculate the minimum water content required to maintain pore fluid continuity within the soil, as well as the corresponding pore size distribution. This study provides a theoretical basis for understanding the linkage between soil conductivity and microstructure. Although there have been many studies on the influencing factors of soil conductivity, further understanding of the complex relationship between moisture content and pore structure on conductivity and its underlying mechanisms still needs to be further explored.

Archie [30] studied the relationship between the resistivity of soil and its structure through experiments, proposed a theoretical model of resistivity suitable for saturated non-cohesive soil, and established the relationship between the resistivity of saturated non-cohesive soil and the porosity of soil. Waxman and Smits [31] established a resistivity model of unsaturated cohesive soils by assuming that the conductivity of soil is an integral conductivity model composed of two conductors in parallel, soil particles and pore water. The model proposed by David Huntley [32] reflects that soil conductivity is the result of the combined effects of soil particle resistivity, pore fluid resistivity, and matrix resistivity. Mitchell [2,33] suggested that soil resistivity is composed of the resistivities of the solid, liquid, and gas phases and proposed a ternary structural model for soil resistivity. Shan et al. [34] derived and established a theoretical model of resistivity by analyzing the effects of initial moisture content, soil temperature and compaction on soil resistivity. Among the numerous empirical and theoretical models mentioned above, some involve a large number of parameters with complex calculations, which are sometimes difficult to estimate accurately, making their application relatively cumbersome. There is a lack of a simple generalized model to assist engineers in understanding the conductivity characteristics of soil, making it convenient and efficient for engineering applications and numerical calculations. In addition, the study of soil conductivity and the prediction model of resistivity in silty regions are also quite limited. In conclusion, there are still relatively few studies that consider the influence of multiple factors on soil conductivity. Moreover, the conduction mechanisms under the interaction of factors such as water content and pore structure remain insufficiently explored in a systematic manner.

Therefore, this paper systematically studies the resistivity variation law of silt clay under different moisture content and dry density conditions by conducting two-phase resistivity tests on remolded silt clay samples. The combined effects of moisture content and pore structure on soil electrical conductivity were analyzed by the nuclear magnetic resonance (NMR) results of silt clay samples. NMR is a non-destructive testing method that preserves the original structure of the sample. With its high sensitivity and multi-parameter linkage capability, it can accurately measure soil pore structures and precisely distinguish different forms of water, providing strong support for in-depth studies on the mechanisms of soil electrical conductivity. The effective contact area between soil particles, the type and quantity of conductive paths were discussed under different moisture content and dry density conditions. The mechanism of three conductive paths between soil particles, pore fluid and soil-water coupling in unsaturated silt clay was clarified. A simple two-parameter generalized calculation model based on experimental data and theoretical analysis is proposed, which provides a scientific basis for the engineering application and numerical calculation of resistivity method in geotechnical engineering.

## Materials and methods

### Basic physical properties of silt clay soil

**Fig 1:**
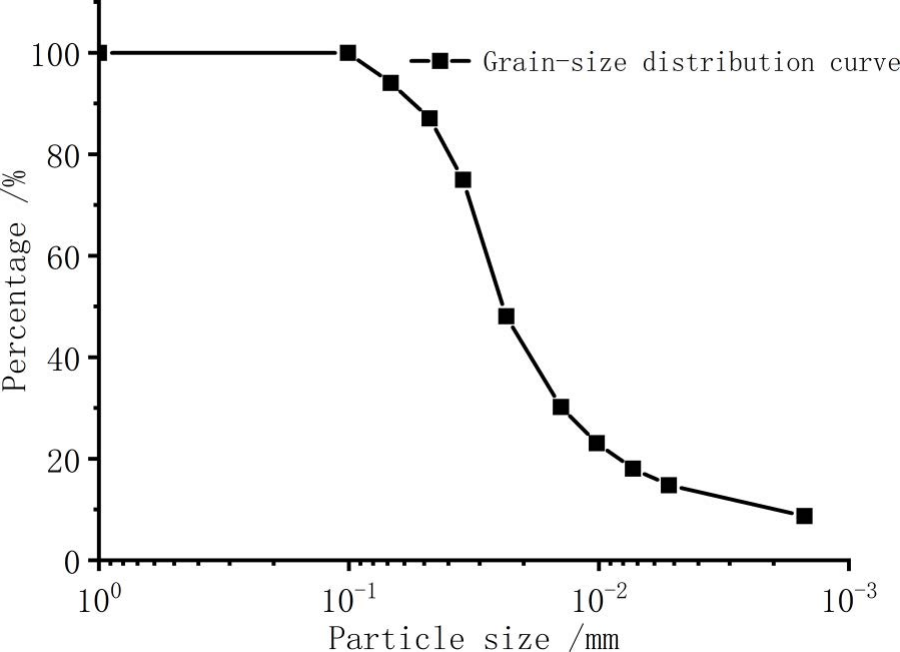
Grain-size distribution curve.

The test soil sample was taken from a foundation pit construction site in Tongzhou District, Beijing, China. As shown in Fig 1, the proportion of particles larger than 0.075 mm was 4.3%. It can be seen from Table 1 that the plasticity index is 6.8%. According to the specific classification of fine-grained soil in the American ASTM standard [35], the test soil in this paper is determined as silty clay. Mineral composition analysis was conducted using a PW4400 X-ray fluorescence (XRF) spectrometer from Panalytical, Netherlands, equipped with an ultra-sharp end-window Rh target X-ray tube and a 75 μm ultra-thin beryllium window. The X-ray tube current was set to 40 mA, and the voltage was set to 40 kV. The diffractometer’s scanning angle (2θ) ranged from 1° to 10° for low-angle scans and from 5° to 70° for high-angle scans. The mineralogical properties of silty clay were measured by X-ray diffraction (XRD) test as shown in Table 2.

**Table 1:** The physico-chemical property of silt clay.

Sample Property	
	Silt clay
Liquid limit	23.3%
Plastic limit	16.5%
Plasticity index	6.8
Specific gravity	2.72
Specific surface area	35m^2^/g
Sand (2-0.075mm)	4.3%
Silt (0.075-0.005mm)	80.3%
Clay (≤0.005mm)	15.4%

**Table 2:** Mineralogical properties of silt clay.

SampleProject	
	Silt clay
Quartz	51.9%
Potassium feldspar	2.2%
Plagioclase	8.7%
Calcite	13.1%
Dolomite	1.6%
Hornblende	2.1%
Illite	9.0%
Kaolin	2.4%
Chlorite	4.1%
Eamonn mixed layer	4.9%

The test methods of the above physico-chemical properties are referenced in [36]. The plastic limit and liquid limit are determined using the combined test method by measuring the penetration depth of a cone into the soil at different moisture contents. The specific gravity is measured using the pycnometer method, the specific surface area is determined through the gas adsorption BET method, and the particle size distribution curve is obtained via the sieving method.

### Test plan

In this paper, the influence of moisture content and dry density on the resistivity of the specimen is studied by configuring compaction samples with different moisture content and different dry densities, and the two-stage resistance measurement is carried out through the digital bridge, and the particular test methods are as follows.

**Fig 2:**
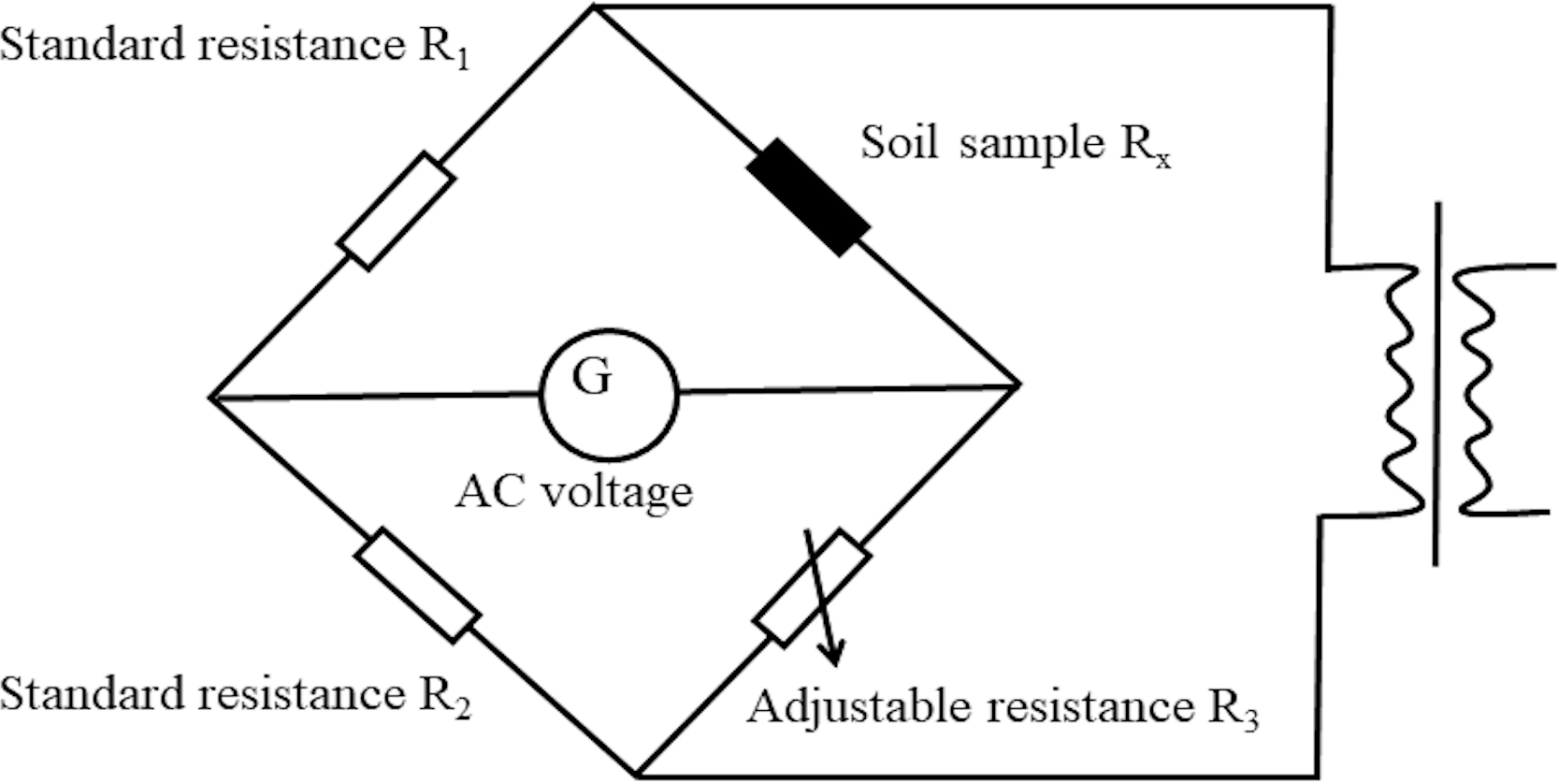
Schematic diagram of digital bridge resistance test structure.


(1) Sample preparation: The soil used in the test was air-dried pulverized, and passed through a 2mm sieve. The moisture content of the soil air-dried under ambient temperature conditions was 3.03%. According to the target moisture content, distilled water of different masses was sprayed into the loose soil, and the fresh-keeping bag was sealed and placed in a moisturizing cylinder for moisturizing and stuffing for 24 hours. The moisture content is remeasured to determine its true value, and the compaction samples are prepared based on the calculated moisture content. After remeasurement, the values were 3.0, 6.5, 10.2, 13.8, 17.2, 20.5, and 23.9%, respectively. The ring knife specimen with a diameter of 61.8mm and a height of 20mm was prepared by static compaction, and the dry density was set to 1.45, 1.55 and 1.65g/cm^3^ respectively, and the sample was wrapped with plastic wrap after being pressed and placed in a moisturizing tank for 24 hours, and the resistance test was carried out after completion.
(2) Resistivity test: The resistance of the soil was measured by the low-frequency AC two-phase electrode method using a VC4091C ICR (Inductance Capacitance Resistance) digital bridge with a measurement frequency of 50 Hz [37]. The resistance measurement principle of the LCR digital bridge is shown in Fig 2, and the measurement range of the bridge is 0.0001 ~  99.9999 MΩ. The process of measuring soil resistance involves covering circular copper plates as electrode plates on the upper and lower surfaces of the ring cutter sample, and adding a negative 500g weight to the upper surface of the ring cutter sample to ensure close contact between the electrode plates and the soil sample. The wires on the electrode plates are connected to the ICR digital bridge, and the average value of the resistance is taken as the measurement result after multiple measurements, the resistivity is calculated as shown in Equation (1).

$$\rho =R\frac{S}{L}$$ (1)

In the formula, *ρ* is resistivity, Ω⋅m. *R* is resistance measured by digital bridge, *Ω*_._ *S* is contact electrode area for two-pole method measurement, $m^{2}$. *L* is the height of specimen, *m*_._

**Fig 3:**
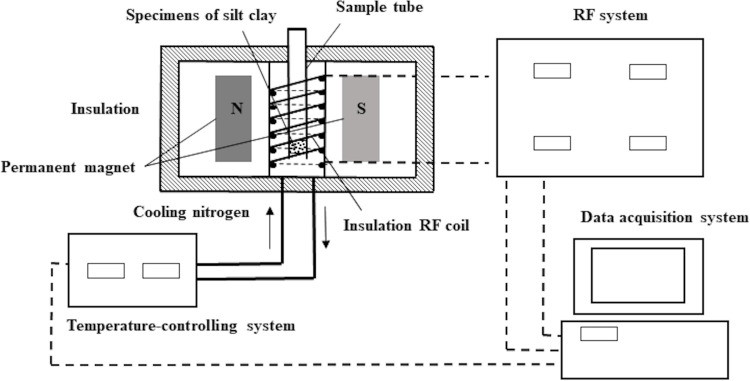
Schematic diagram of the structure of the nuclear magnetic test system.

(3) NMR: The dry density and water content of the nuclear magnetic resonance (NMR) samples are consistent with those used for resistivity testing. However, the NMR samples are prepared in plastic ring molds with a diameter of 40 mm and a height of 20 mm, and the NMR experiments are conducted at room temperature. The PQ-001 Mini-NMR device was utilized to monitor the evolution of the *T*_2_ transverse (spin-spin) relaxation time distributions, which were analyzed to explore the pore water distribution in samples. The measurements were carried out using a magnetic field intensity of 0.52 Tesla and a frequency of 23.0 MHz. The Carr-Purcell-Meiboom-Gill (CPMG) pulse sequence was implemented, with an echo time set at 36 µs and 2000 echoes within the sequence. NMR signals were used to obtain the induction decay curve, which was then inverted using a Fourier transform to yield the final *T*_2_ distribution. The structure diagram of the nuclear magnetic test is shown in Fig 3.

## Results

### Effect of moisture content

**Fig 4:**
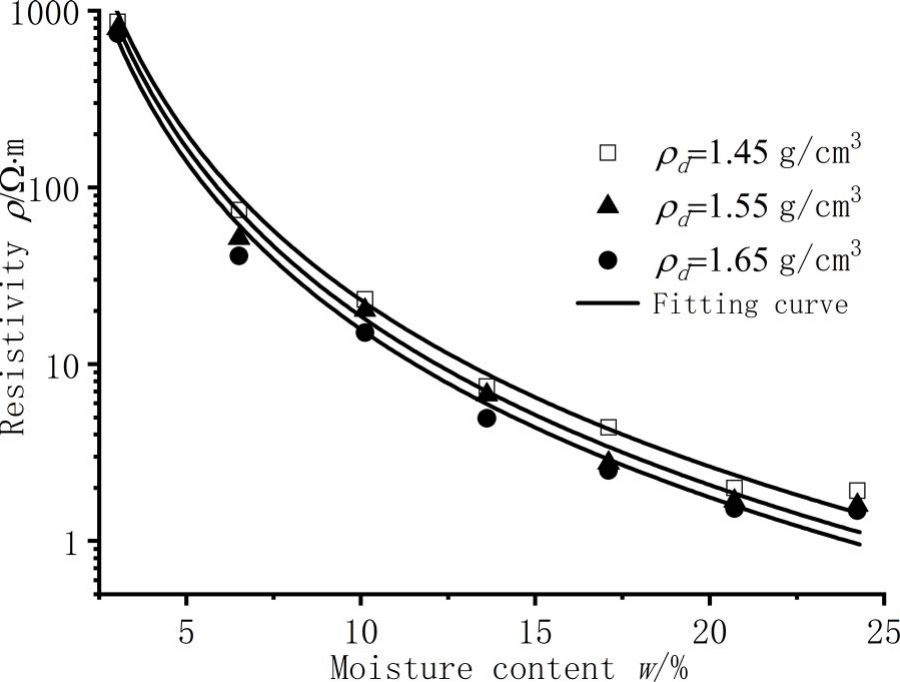
Resistivity of silt clay under different moisture content.


Fig 4 is the resistivity test results of silt clay under different moisture content conditions, and according to the law of the test results, the resistivity is obtained by function fitting as a power function of moisture content, as shown in Equation (2). Table 3 shows the parameter results of function fitting, and the fitting degree R^2^ is above 0.98.

**Table 3:** Parametric results of function fitting.

Dry density/g/cm^3^	*a*	*b*	R^2^	P	Significance/Not significance(S/NS)
1.45	0.0171	-3.131	0.99	<0.001	S
1.55	0.0126	-3.170	0.99	<0.001	S
1.65	0.0110	-3.157	0.98	<0.001	S

$$\rho =aw^{b}$$ (2)

In the formula, *ρ* is resistivity, Ω⋅m. *w* is moisture content, dimensionless no unit. *a, b* are the material parameters obtained by fitting, dimensionless no unit.

As shown in Fig 4, the lower the moisture content, the greater the resistivity, and with the increase of the moisture content, the resistivity begins to drop sharply in the low moisture content section, from 400 ~ 600 Ω to 20 ~ 60 Ω⋅m. With the continuous increase of moisture content, the change in resistivity is no longer significant and gradually stabilizes at around 1 Ω⋅m. The optimal moisture content of the silt clay used in this article is *w* = 14.5%, and the resistivity of the silt clay begins to stabilize around the optimal moisture content. The trend of silt clay resistivity test results obtained in this study is consistent with the research results of other scholars [12,13].

### Effect of dry density

**Fig 5:**
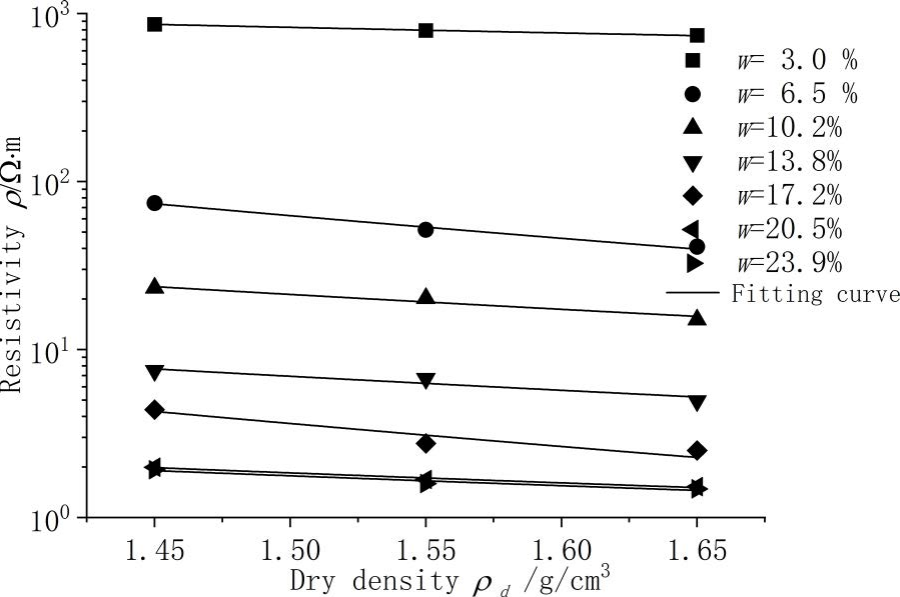
Resistivity of silt clay under different dry densities.


Fig 5 shows the resistivity test results of silt clay under different dry density conditions. According to the rules of the test results, it is known that the resistivity has a good fitting result through the power function relationship through function fitting, as shown in formula (3). Table 4 shows the parameters of the function fitting, the fitting degree R^2^ is above 0.90, and the fitting result is good.

**Table 4:** Parametric results of function fitting.

Moisture content/%	*a*	*b*	R^2^	P	Significance/Not significance (S/NS)
3.0	1341.4	-1.19	1.00	0.003	S
6.5	409.0	-4.635	0.99	0.022	S
10.2	82.88	-3.344	0.95	0.034	S
13.8	25.40	-3.194	0.92	0.043	S
17.2	21.11	-4.374	0.90	0.065	NS
20.5	4.28	-2.078	0.98	0.012	S
23.9	4.02	-2.028	0.94	0.022	S

$$\rho =a\rho_{d}^{b}$$ (3)

In the formula, *ρ* is resistivity, Ω⋅m. *a*, *b* are the material parameters obtained by fitting, dimensionless no unit. *ρ*_d_ is the dry density of soil, g/cm^3^.

According to Fig 5, regardless of the moisture content conditions, the higher the dry density of the silt clay sample, the smaller the resistivity. As the dry density increases, the resistivity continuously decreases. With the increase of the moisture content of the sample, the resistivity increase rate of the sample gradually flattens when the dry density of the sample increases, and the change amplitude of parameter a in Equation (3) is not obvious. When the dry density increases near the maximum dry density, the increase in dry density has little effect on the change in resistivity, as shown in Fig 5, and the resistivity measurements for the two dry densities are intensely similar. Therefore, the effect of dry density of silt clay samples on resistivity is smaller than the effect of moisture content on resistivity.

### ^1^H-NMR

**Fig 6:**
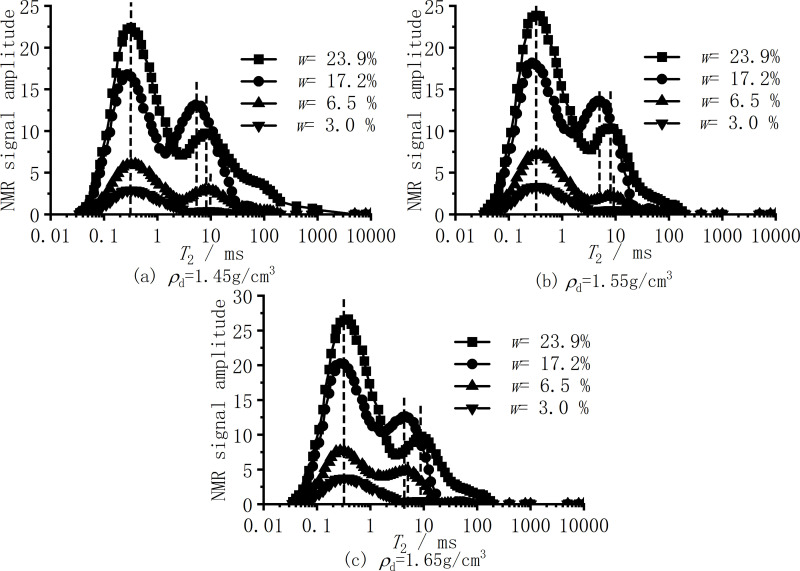
*T*_2_ curve distribution of samples with different dry densities. (a) *ρ*_*d*_ = 1.45g/cm^3^ (b) *ρ*_*d*_ = 1.55g/cm^3^ (c) *ρ*_*d*_ = 1.65g/cm^3^.

Fig 6 shows the NMR curves of samples with different dry densities under four different moisture contents of silt clay. As shown in Fig 6, the NMR curves of all samples show a bimodal structure. The relaxation time of the peak center of the left peak is about 0.3 ms, while the relaxation time of the peak center of the right peak is about 4–10 ms.

**Fig 7:**
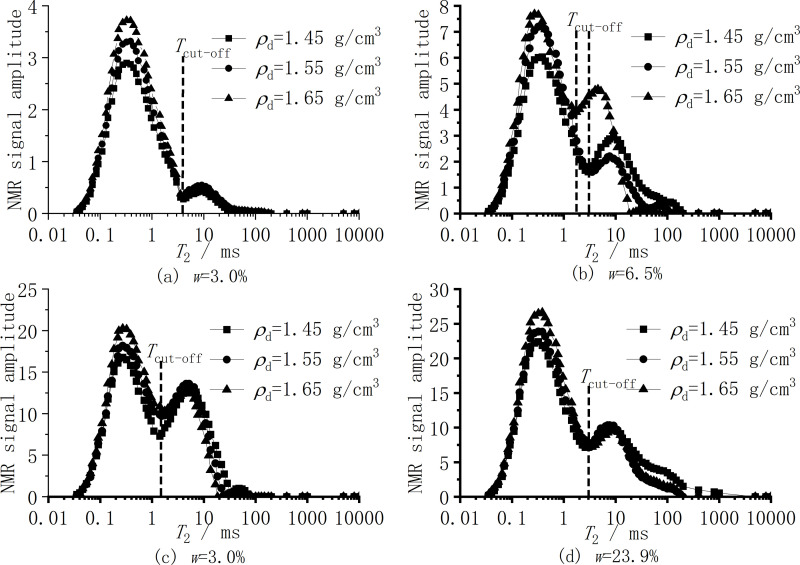
*T*_2_ curve distribution of samples with different initial moisture contents. (a) *w* = 3.0% (b) *w* = 6.5% (c) *w* = 17.2% (d) *w* = 23.9%.

Fig 7 shows the NMR curves of samples with different moisture contents under three different dry densities of silt clay. As shown in Fig 6, there is a slight difference in the cut-off threshold between the two peaks of samples with different moisture contents under three dry densities. The cut-off threshold value of samples with a moisture content of *w* = 3.0% is relatively consistent at 3.5ms, while the cut-off threshold value of samples with a moisture content of *w* = 6.5% is between 1.9ms and 3.0ms. The cut-off threshold values of samples with a moisture content of *w* = 17.2% and *w* = 23.9% are about 1.5ms and 3.0ms, respectively. The cut-off threshold of the specific nuclear magnetic test is shown in Table 5.

**Table 5:** The cut-off threshold for different samples.

Dry density/g/cm^3^Moisture content/% Cut-off value/ms			

	1.45	1.55	1.65
3.0	3.5	3.5	3.5
6.5	1.9	3.0	3.0
17.2	1.5	1.5	1.5
23.9	3.0	3.0	3.0

The NMR spectrum in Fig 6 shows that the area and shape of the obtained spectrum will gradually increase with the increase of moisture content due to the large difference in moisture content, so that the peak shape will not overlap. Fig 7 is the NMR spectrum of different dry densities under the same moisture content of the soil. In Fig 6a, because the moisture content is only 3.0% and the moisture content is very low, the increase of dry density is quantitatively manifested in the increase of bound water. The reaction on the NMR spectrum is that the amplitude of the left peak is increasing, while the amplitude of the right peak is very small. The amplitude of the right peak curves for the three dry density specimens differs by no more than 0.5, indicating that the right peaks of the three dry densities are essentially coincident. Fig 7b through Fig 7d show that an increase in dry density and a continuous increase in total moisture content correspond to an increase in the relaxation range and amplitude of the right peak. The right relaxation range for samples with lower dry density under the same moisture content is greater than that for samples with higher dry density. This is because as the dry density increases, soil porosity decreases, leading to a reduction in the content of larger pores. Consequently, the area of the small-pore peak, represented by the left peak on the NMR spectrum, increases. Therefore, the right peaks in Fig 7c and 7d show minor differences and are nearly coincident.

In Fig 7b, the right peak of *ρ*_*d* = _1.45g/cm^3^ is similar to that of *ρ*_*d* = _1.55g/cm^3^, and the difference of *ρ*_*d* = _1.65g/cm^3^ is because when the water content increases to *w* =  6.5%, the pore water begins to appear, and the resistivity begins to decrease rapidly as shown in Fig 4. The pore water content is insufficient, and with the increase in dry density, some water migrates to the mesopores and falls between the left and right peaks. On the other hand, the smaller the amplitude of the Y-axis coordinate is, the larger the difference looks, while there is no difference from Fig 7a.

## Discussion

**Fig 8:**
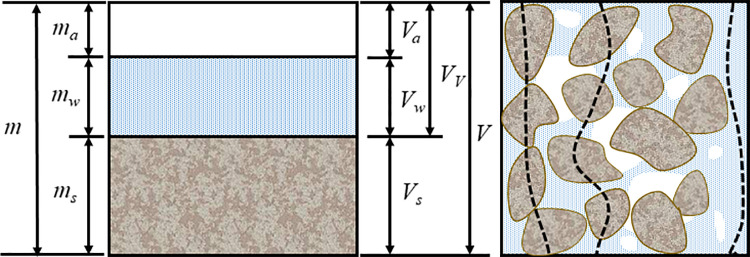
Schematic diagram of the three phases and conductive paths of soil: *m* is the total mass of soil, *m*_a_ is the gas mass, *m*_*w*_ is the pore liquid mass, *m*_*s*_ is the solid mass, *V* is the total volume of soil, *V*_v_ is the pore volume, *V*_a_ is the gas volume, *V*_w_ is the pore liquid volume, *V*_s_ is the solid volume.

As shown in Fig 8, there are mainly soil (S), water (W), and air (A) three phases in the unsaturated soil, because the resistivity of the air is much larger than that of the soil and water, it is generally considered to be non-conductive, and only soil particles and pore water are conductive in the soil at this time. Therefore, there are three main conductive paths between unsaturated soils, and the conductive paths represented by the three dashed lines arranged in turn in Fig 8 are the conductive paths between pore water, soil particles, and soil-water coupling [2,33].

The conductive path between soil particles is usually an insulator or semiconductor, which is a common soil component such as silicate minerals and alumina minerals. Therefore, the conductive ability between soil particles is weak, mainly relying on a small amount of conductive ions in minerals for limited current conduction. The electrical conductivity between soil and water refers to the participation of the water film adsorbed on the surface of soil particles in conductivity. Changes in moisture content can lead to changes in the thickness of water adsorbed on the surface of soil particles. The thickness of the water film determines the unit amount of ions conducting current. The electrical conductivity of soil-water coupled paths is closely linked to moisture content and is moderate compared to the other two types of conductive paths. The primary component contributing to conductivity in the pore water conductive path is capillary water, which flows freely within soil pores, and contains a high concentration of dissolved ions [29,33]. The electrical conductivity of pore water paths mainly depends on the ion concentration within the pore water and the connectivity of the pores, making pore water the most conductive. Under different moisture content and dry density conditions, the types and number of active conductive paths vary, leading to differences in resistivity. The following analysis will explore and verify these relationships using moisture content, dry density, and ^1^H-NMR test results.

### ^1^H-NMR analysis

In unsaturated porous media, due to the strong interaction between the surface of solid particles and pore water, the relaxation time of nuclear magnetic resonance can be understood as the interaction time between water molecules and the surface of soil particles. Therefore, the shorter the relaxation time *T*_2_, the closer the water molecules are to the surface of soil particles. Adsorbed water or water in small pores relaxes faster than water in free water or large pores, resulting in a smaller *T*_2_ value [38–40]. Therefore, the left part of the cut-off threshold of the *T*_2_ distribution curve is adsorbed water, while the right side corresponds to capillary water, which can be divided into different water occurrence states of *T*_2_ measured by NMR. In saturated porous media, water in large pores can be defined as free water. For dense small pores, because the pore size is relatively small and the pore water is mainly adsorbed water, pores smaller than a certain size are all defined as bound water. In the distribution curve of the pore structure, Romero et al. [41] and Wang et al. [42] used the trough as the cut-off point of the pore structure. The following text refers to this method of dividing pore structure to define the occurrence state of pore water.

Tian et al. [43] quantitatively analyzed the moisture state of soil during freeze-thaw cycles, and determined the matrix suction by setting *T*_2_ = 5.8ms as the threshold to divide unfrozen pore water into adsorbed and capillary states. Jaeger et al. [44] determined the *T*_2_ range of adsorbed water on frozen peat soil and found that the relaxation range of the cut-off threshold value for adsorbed water was 0.9ms < *T*_2_ < 1.1ms. Ma et al. [45] studied the microstructure evolution of expansive soil during the dry-wet cycle, and determined that the threshold of pore water adsorption and capillary state was *T*_2_ = 0.86ms. Different soil samples will have different pore distribution and water adsorption due to differences in soil mineral composition and specific surface area, which is manifested macroscopically as differences in the cut-off threshold between adsorption water and capillary water. The cut-off values of silt clay under different moisture contents in this article are all within the threshold range defined by the above research results. Therefore, referring to the current research results, the cut-off value of the sample when the moisture content is the largest in the middle of the cut-off value of all moisture content samples is taken as the threshold, that is, *T*_cut-off_ = 3.0ms is the cut-off value of adsorbed water and capillary water.

### The influence of moisture content and dry density on resistivity

The changes in electrical conductivity caused by changes in moisture content can be attributed to factors such as water film thickness, continuity of conductive paths, and distribution of moisture in soil particles and pores [2]. Small pore size *d* = 5μm limits the movement of water and electrolytes [45], resulting in poor conductivity and high resistivity. Large pore size can accommodate more water and electrolytes, improve conductivity, and thus reduce resistivity. The specific analysis of the electrical conductivity of silt clay under different moisture content conditions is as follows.

1) In the low moisture content stage (*w* < 3.0%), the surface of the soil particles of silt clay has a negative charge, and water molecules are adsorbed on the surface of the soil particles. As shown in Fig 5a, the sample with *w* < 3.0% only contains adsorbed water, and there is no capillary water between the soil. At this time, the water film on the surface of the soil is bound water with strong adsorption. The conductive path in the soil is mainly between soil particles and part of the water film contacts. Meanwhile, the contact area between soil particles is small, and the main conductive path relies on a small amount of conductive ions in the silt clay minerals for limited current conduction. In the case of low moisture content, water will be unevenly distributed in the pores, and there may be drying points in certain areas, causing the conductivity path to be interrupted [44,45]. The soil has a lower conductivity and a higher resistivity.
2) At the stage of moderate moisture content (3.0% ≤ *w* ≤ 17.2%), as shown in Fig 6, with the increase of moisture content, the area of the left peak of *T*_cut-off_ = 3.0ms in the NMR spectrum gradually increases, while the right peak begins to appear and the area gradually increases. That is, After the water film on the surface of soil particles continuously thickens, on the one hand, the water in the soil thickens from adsorbed bound water to capillary water, which can flow freely and form a liquid bridge between soil particles, forming a soil-water coupling conductive path. As the moisture content increases, capillary water forms a pathway, increasing the conductive path of pore fluid. On the other hand, a certain amount of soluble salts naturally present in the soil gradually dissolve into the pore solution as the moisture content increases, thereby increasing the ion concentration in the pore solution [2,33]. Due to the polarization characteristics of hydrated ions and their mobility in the electric field of the solution, the electrolyte exhibits high conductivity [47,48]. Relevant studies have shown that the clay mineral composition can further increase the number of conductive ions in the pore solution through mechanisms such as isomorphous substitution or hydrolysis [33]. As indicated in Table 2, the mineral composition of the silt clay used in this study contains a certain proportion of clay minerals. Therefore, under the combined effects of soluble salts and clay minerals, the resistivity of the silt decreases sharply within this range of moisture content.
3) In the high moisture content stage (w > 17.2%), as shown in Fig 6, after the moisture content increases to a certain stage, although the right peak area of *T*_cut-off_ = 3.0ms in the NMR spectrum continues to increase, mainly within a larger relaxation range. Such as the change in the conductive path that occurs at a moisture content of 3.0% ≤ *w* ≤ 17.2% is no longer obvious. On the other hand, the increase in moisture content enhances the continuity of capillary water and enhances its conductivity. The number of ions contributed by factors such as soluble salts is limited. Beyond a certain moisture content, no additional salt ions will dissolve into the pore solution. As the moisture content continues to increase, the concentration of salt ions in the pore solution will undergo dilution and decrease accordingly, leading to a decrease in conductivity [29,47]. The final effect is to gradually stabilize the resistivity. Therefore, the increase of water content in this interval will not cause a significant change in resistivity, which explains the nearly overlapping results of the resistivity results of *w* = 20.5% and *w* = 23.9% under different dry densities in Fig 3. The resistivity has begun to stabilize when the soil is near the optimal moisture content. Based on this, in engineering applications, the optimal moisture content of the soil can be quickly measured by measuring the moisture content when the resistivity begins to stabilize without conducting standard compaction tests. Similarly, Fukue et al. [29] determined the boundary moisture content of the soil by measuring the resistivity.

As shown in Fig 7, the size of the dry density determines the pore volume (*V*_v_) and soil particle volume (*V*_s_). The larger the dry density of the sample, the larger the space occupied by the soil particles, the smaller the relative pore volume, the larger the effective contact area of the soil particles, and the lower the resistivity [29,33]. As shown in Fig 5, under the same moisture content conditions, the NMR spectrum does not change much when the soil dry density changes. The increase in soil mass leads to an increase in the moisture content of the sample. The corresponding peak shape and relaxation range in the NMR spectrum do not change, but increase slightly in amplitude. On the pore structure under the same water content, the small pores of the sample with large dry density increase and the large pores decrease, which increases the effective contact area of the conductive path between soil particles, and increases the probability and number of pore water forming liquid bridges between soil particles, so that the soil-water coupling and pore water conductive path increase, and finally the soil resistivity decreases [2,29,33,46]. Higher dry density tends to mean tighter particle arrangement and more uniform pore distribution, which helps to form a stable conductive path, increase the continuity of the conductive path, and reduce resistivity.

However, the impact of changes in dry density on the resistivity of silt clay is not as significant as changes in moisture content. On the other hand, in the three-phase system of soil, the electrical conductivity of the solid phase is much weaker than that of the liquid phase [29,33]. Overall, this is because silt clay is a water-sensitive mineral soil, and moisture content significantly affects the resistivity of soil by changing the continuity of water film and ion conduction path, while the impact of dry density is indirect and relatively small. Therefore, in the study of resistivity of silt clay, the moisture content of the soil has a more significant decisive role than the dry density [2,45,46].

### Model calculation

**Fig 9:**
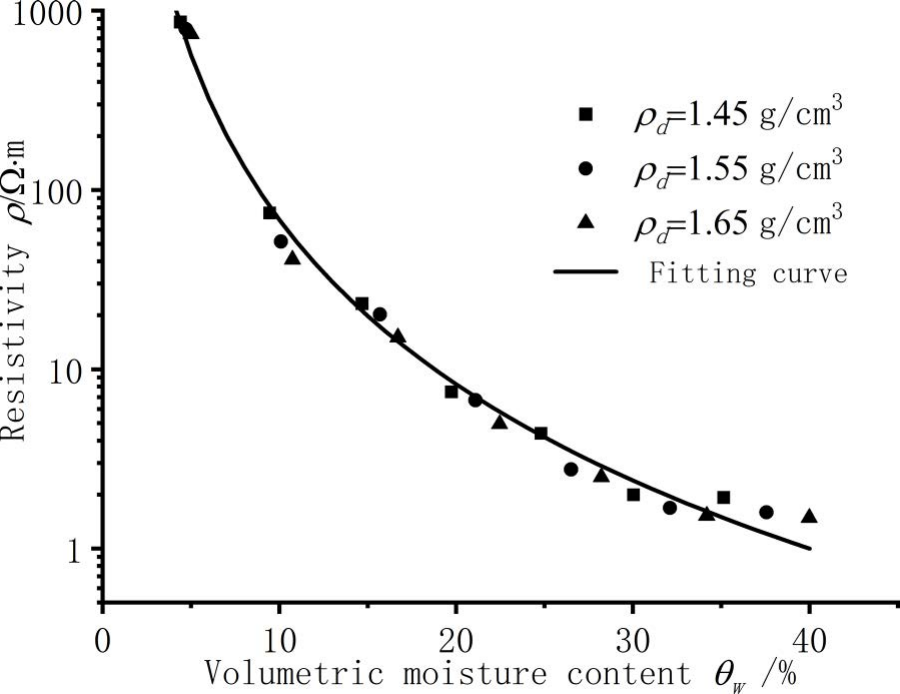
Resistivity of silt clay under different volume moisture content conditions.

**Fig 10:**
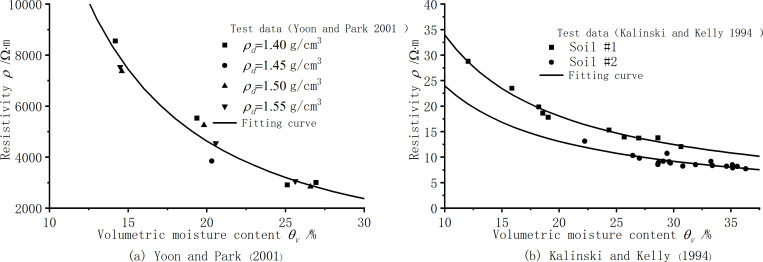
Model verification of different soil resistivity. **(a)** Yoon and Park (2001) **(b)** Kalinski and Kelly (1994).


In engineering practice, the two parameters of soil moisture content and dry density cannot be separately discussed for their impact on soil resistivity. These two key influencing factors together determine the magnitude of soil resistivity. To comprehensively consider the impact of these factors on soil resistivity and facilitate its application in engineering, it is necessary to establish a resistivity model that comprehensively considers soil dry density and moisture content. Such a model will more comprehensively reflect the complex changes in soil resistivity and provide more targeted guidance for engineering design. By introducing volumetric moisture content to comprehensively consider the influence of soil moisture content and dry density on resistivity, the calculation method of volumetric moisture content *θ*_w_ is shown in formula (4), and the corresponding resistivity model is shown in formula (5). Fig 9 shows the resistivity test results of silt clay, and Fig 10 shows the resistivity test results of Yoon and Park [4] on sandy soil and Kalinski and Kelly [21] on two kinds of clay under simulated engineering site conditions. The model in this paper can simulate these results well, and the difference between the soil #2 simulation is that the results of the experimental data are more discrete. The results of the model parameters are shown in Table 6.

**Table 6:** Parameter results of the model.

Soil type	*a*	*b*	R^2^	P	Significance/Not significance(S/NS)
silt clay (in this study)	76242	-3.048	0.99	<0.001	S
Yoon and Park (2001)	648579	-1.65	0.96	<0.001	S
Kalinski and Kelly (1994) -Soil #1	277.1	-0.912	0.98	<0.001	S
Kalinski and Kelly (1994) -Soil #2	179.33	-0.874	0.85	<0.001	S

$$\theta_{w}=\frac{V_{w}}{V}=\frac{w\rho_{d}}{\rho_{w}}$$ (4)

$$\rho =a\theta_{w}^{b}$$ (5)

In the formula, *ρ* is resistivity, *θ*_w_ is the volumetric water content of the soil, dimensionless no unit. *V*_w_ is the volume of water, cm^3^. *V* is the total volume of the soil, cm^3^. *w* is the moisture content of soil, dimensionless no unit; *ρ*_d_ is the dry density of soil, g/cm^3^. *ρ*_*w*_ is the density of water, *ρ*_*w*_ = 1.0 g/cm^3^. *a*, *b* are the material parameters obtained by fitting, dimensionless no unit.

As shown in Fig 9, in the absence of other factors, soil typically exhibits a unique volumetric moisture content-resistivity relationship curve, which is less affected by other factors. In practical engineering applications, by establishing a volume moisture content conductivity model, the two-phase electrode method can be used to non-destructive and rapid determine the soil resistivity, and thus quickly determine the volume moisture content of the soil. This method is efficient and accurate, providing convenience for rapid evaluation of soil properties.

## Conclusions

In this paper, the resistivity of silt clay under different moisture content and dry density is measured by the two-stage method, and the influence mechanism of water state and pore structure on the resistivity is explored by using NMR assisted analysis. The main conclusions are as follows:

Resistivity experiments on silt clay with varying initial moisture content and dry density indicate that resistivity decreases sharply with increasing moisture content and then gradually stabilizes. It also decreases progressively with higher dry density. The sensitivity of soil resistivity to moisture content is significantly greater than its sensitivity to dry density, with resistivity stabilizing near the soil’s optimal moisture content.

NMR test results reveal that the cut-off value between adsorbed water and capillary water is *T*_cut-off_ = 3.0ms. The soil’s moisture state determines the number and efficiency of conductive paths between pore water, soil particles, and the soil-water coupling system. Dry density influences the pore structure, modifies the effective contact area along conductive paths between soil particles, and affects pore connectivity, collectively impacting soil resistivity.

Without considering other influencing factors, the silt clay sample exhibits a unique volumetric moisture content-resistivity curve, where volumetric moisture content and resistivity are uniquely correlated through a power function relationship model. In engineering practice, the volumetric moisture content of soil can be rapidly determined using resistivity measurements, providing a valuable reference for ensuring the safety of construction and operational processes. Future research should refine the volumetric moisture content-resistivity model by integrating factors like soil mineralogy and electrochemical properties and validating it across various silt clay samples and field conditions.

## References

[1] Jardine RJ. Geotechnics, energy and climate change: the 56th Rankine Lecture. Géotechnique. 2020;70(1):3–59.

[2] Osman SBS, Fikri MN, Siddique FI. Correlation of electrical resistivity with some soil parameter for the development of possible prediction of slope stability and bearing capacity of soil using electrical parameters. Pertanika Journal of Science & Technology. 2012;22(1):139–52.

[3] G. Y, M. O, J. P. Laboratory study of landfill leachate effect on resistivity in unsaturated soil using cone penetrometer. Environmental Geology. 2002;43(1–2):18–28. doi: 10.1007/s00254-002-0649-1

[4] Yoon GL, Park JB. Sensitivity of leachate and fine contents on electrical resistivity variations of sandy soils. J Hazard Mater. 2001;84(2–3):147–61. doi: 10.1016/s0304-3894(01)00197-2 11406303

[5] Richard G, Séger M, Besson A. Resistivity to assess soil properties[M]. Berlin: Springer Netherlands; 2014.

[6] Qazi W, Memon MB. Effects of shear strength properties on resistivity of compacted laterite soil: a conceptual model[C]. Malaysian Universities Conference on Engineering and Technology. Malaysia; 2015.

[7] Asif A, Ali SS, Noreen N. Correlation of resistivity of soil with geotechnical engineering parameters at Wattar area district Nowshera, Khyber Pakhtunkhwa, Pakistan. Journal of Himalayan Earth Sciences. 2016;49(1):124–30.

[8] Wychowaniak D, Zawadzki Ł, Lech M. Application of column tests and electrical resistivity methods for leachate transport monitoring. Annals of Warsaw University of Life Sciences, Land Reclamation. 2015;47(3):237–47. doi: 10.1515/sggw-2015-0028

[9] Osman SBS, Fikri MN, Siddique FI. Correlation of electrical resistivity with some soil parameter for the development of possible prediction of slope stability and bearing capacity of soil using electrical parameters. Pertanika Journal of Science & Technology. 2012;22(1):139–52.

[10] Li Q, Cheng YF, Li Q. Establishment and evaluation of strength criterion for clayey silt hydrate-bearing sediments. Energy Sources, Part A: Recovery, Utilization, and Environmental Effects. 2018;40(1–6):742–50. doi: 10.1080/15567036.2018.1431234

[11] Li Q, Wang YL, Wang FL. Effect of thickener and reservoir parameters on the filtration property of CO2 fracturing fluid. Energy Sources, Part A: Recovery, Utilization, and Environmental Effects. 2020;42(13/18):1705–15.

[12] Lu Y, Cai G, Zhao C. The shear strength of granite weathered soil under different hydraulic paths. Applied Sciences. 2020;10(18):6615.

[13] Ferre PA, Redman JD, Rudolph DL. The dependence of the electrical conductivity measured by time domain reflectometry on the water content of a sand. Water Resources Research. 1998;34(5):1207–13.

[14] Saarenketo T. Electrical properties of water in clay and silty soils. Journal of Applied Geophysics. 1998;40(1–3):73–88. doi: 10.1016/s0926-9851(98)00017-2

[15] Ahmed AM, Sulaiman WN. Evaluation of groundwater and soil pollution in a landfill area using electrical resistivity imaging survey. Environ Manage. 2001;28(5):655–63. doi: 10.1007/s002670010250 11568845

[16] Hassona F, Hassan M, Abu-Heleika M. Correlations between resistivity and some properties of clayey soil. The 3rd International Conference on Site Characterization, ISC’3. 2008 837–41.

[17] Siddiqui FI, Osman SBABS. Simple and multiple regression models for relationship between electrical resistivity and various soil properties for soil characterization. Environ Earth Sci. 2012;70(1):259–67. doi: 10.1007/s12665-012-2122-0

[18] Samouëlian A, Cousin I, Tabbagh A, Bruand A, Richard G. Electrical resistivity survey in soil science: a review. Soil and Tillage Research. 2005;83(2):173–93. doi: 10.1016/j.still.2004.10.004

[19] Long M, Donohue S, L’Heureux JS. Relationship between electrical resistivity and basic geotechnical parameters for marine clays. Canadian Geotechnical Journal. 2012;49(10):1158–68. doi: insert_doi_here

[20] McCarter W. The resistivity characteristics of compacted clays. Geotechnique. 1984;34(2):263–7.

[21] Kalinski R, Kelly W. Electrical-resistivity measurements for evaluating compacted-soil liners. Journal of Geotechnical Engineering. 1994;120(2):451–7.

[22] Abu-Hassanein ZS. Use of resistivity measurement as a quality control tool for compacted clay liners. n.d. .

[23] Abu-Hassanein Z, Benson C, Blotz L. Electrical resistivity of compacted clays. Journal of Geotechnical Engineering. 1996;122(5):397–406.

[24] McNeill JD. Use of electromagnetic methods for groundwater studies. Geotech and Geophys. n.d.;20(5):191–218.

[25] Christensen NB, Srensen KI. Surface and borehole electric and electromagnetic methods for hydrogeological investigations. European Journal of Environmental and Engineering Geophysics. 1998;3(1):75–90.

[26] Dannowski G, Yaramanci U. Estimation of water content and porosity using combined radar and geoelectrical measurements. European Journal of Environmental and Engineering Geophysics. 1999;4 71–85.

[27] Slater LD, Reeve AS. Investigating peatland stratigraphy and hydrogeology using integrated electrical geophysics. Geophysics. 2002;67(2):365–78.

[28] Garambois S, Sénéchal P, Perroud H. On the use of combined geophysical methods to assess water content and water conductivity of near-surface formations. Journal of Hydrology. 2002;259(1–4):32–48. doi: 10.1016/s0022-1694(01)00588-1

[29] Fukue M, Minato T, Horibe H, Taya N. The micro-structures of clay given by resistivity measurements. Engineering Geology. 1999;54(1–2):43–53. doi: 10.1016/s0013-7952(99)00060-5

[30] Archie GE. The electric resistivity log as aid in determining some reservoir characteristics. Transactions American Institute of Mining Metallurgical and Petroleum Engineers. 1942;146(3):54–61.

[31] Waxman MH, Smits LJM. Electrical conductivity in oil-bearing shaly sand. Society of Petroleum Engineers Journal. 1968;8(2):107–22.

[32] Huntley D. Relations between permeability and resistivity in granular aquifers. Ground Water. 1986;24(4):466–74.

[33] Mitchell J K, Soga K. Fundamentals of soil behavior[M]. John Wiley & Sons New York, 2005.

[34] Shan W, Liu Y, Hu Z. A model for the electrical resistivity of frozen soils and an experimental verification of the model. Cold Regions Science and Technology. 2015;119 75–83.

[35] ASTM D2487-00 Standard Practice for Classification of Soils for Engineering Purposes (Unified Soil Classification System)[S].

[36] CS (Chinese Standard). Standard test methods for soils. SL237-1999. Beijing: China Water Conservancy and Hydropower Press; 1999.

[37] Chu Y, Liu S, Cai G. An experimental study of physical and electrical characteristics of zinc contaminated silty clay. Rock and Soil Mechanics. 2015;36(10):2862–8. (in Chinese)

[38] Bayer JV, Jaeger F, Schaumann GE. Proton Nuclear Magnetic Resonance (NMR) Relaxometry in Soil Science Applications~!2009-05-04~!2010-01-25~!2010-06-18~!. TOMRJ. 2010;3(2):15–26. doi: 10.2174/1874769801003020015

[39] Bird NRA, Preston AR, Randall EW, Whalley WR, Whitmore AP. Measurement of the size distribution of water‐filled pores at different matric potentials by stray field nuclear magnetic resonance. European J Soil Science. 2004;56(1):135–43. doi: 10.1111/j.1351-0754.2004.00658.x

[40] Todoruk TR, Langford CH, Kantzas A. Pore-scale redistribution of water during wetting of air-dried soils as studied by low-field NMR relaxometry. Environ Sci Technol. 2003;37(12):2707–13. doi: 10.1021/es025967c 12854709

[41] Romero E, Vecchia GD, Jommi C. An insight into the water retention properties of compacted clayey soils. Géotechnique. 2011;61(4):313–28.

[42] Wang JD, Li P, Ma YW. Evolution of pore-size distribution of intact loess and remolded loess due to consolidation. Journal of Soils and Sediments. 2019;19(3):1226–38.

[43] Tian H, Wei C, Lai Y. Quantification of water content during freeze–thaw cycles: a nuclear magnetic resonance based method. Vadose Zone Journal. 2017;17(1):1–12.

[44] Jaeger F, Shchegolikhina A, As HV. Proton NMR relaxometry as a useful tool to evaluate swelling processes in peat soils. Open Magnetic Resonance Journal. 2010;3(2):27–45.

[45] Ma T, Wei C, Yao C. Microstructural evolution of expansive clay during drying–wetting cycle. Acta Geotechnica. 2020;15(8):2355–66.

[46] Kalinski RJ, Kelly WE. Electrical‐Resistivity Measurements For Evaluating Compacted‐Soil Liners. J Geotech Engrg. 1994;120(2):451–7. doi: 10.1061/(asce)0733-9410(1994)120:2(451

[47] Klein KA, Santamarina JC. Electrical Conductivity in Soils: Underlying Phenomena. JEEG. 2003;8(4):263–73. doi: 10.4133/jeeg8.4.263

[48] Samouëlian A, Cousin I, Tabbagh A, Bruand A, Richard G. Electrical resistivity survey in soil science: a review. Soil and Tillage Research. 2005;83(2):173–93. doi: 10.1016/j.still.2004.10.004

